# Incomplete synaptic vesicle priming functionally disconnects CA1 pyramidal cells

**DOI:** 10.1126/sciadv.aej3501

**Published:** 2026-11-11

**Authors:** Noemi Holderith, Zoltan Nusser

**Affiliations:** Laboratory of Cellular Neurophysiology, HUN-REN Institute of Experimental Medicine, Budapest 1083, Hungary.

## Abstract

The hippocampal CA1 area is perceived as a nonrecurrent neuronal network, i.e., CA1 pyramidal cells (CA1PCs) within the CA1 area innervate mainly γ-aminobutyric acid–releasing interneurons but not each other. Here, we performed an unbiased examination of the functional properties of local CA1PC output synapses using in vitro two-photon glutamate imaging. Fluorescent transients measured with iGluSnFR3 in individual boutons displayed extraordinary heterogeneity in their amplitudes and short-term plasticity patterns upon a complex train of presynaptic action potential firing. Post hoc electron microscopy of the imaged boutons demonstrated that distinct postsynaptic structure-innervating boutons display different functional properties. Approximately 25% of the boutons innervate CA1PC spines, which either release glutamate with very low release probability or completely fail to release. Phorbol 12,13-dibutyrate and elevated calcium (2+) concentration, manipulations that enhance synaptic vesicle priming and fusion, convert these silent into properly functioning synapses. Our results provide evidence that CA1PCs are interconnected with structurally and molecularly intact glutamatergic synapses, but incomplete synaptic vesicle (SV) priming precludes SV release.

## INTRODUCTION

Although the hippocampal formation is one of the most studied areas in the mammalian brain, there are many neuronal connections in this circuit that are rather unexplored. In the trisynaptic pathway, only the principal cells of the CA3 area form a recurrent network (*1*–*4*), which is thought to underlie computations such as pattern completion (*5*, *6*). The dentate gyrus (DG) and CA1 area are considered as feedforward networks, where the local collaterals of principal cells only innervate local circuit γ-aminobutyric acid–releasing (GABAergic) interneurons (INs) and local circuit glutamatergic mossy cells (in DG only). The lack of monosynaptic connections among DG granule cells is expected because their local axon collaterals avoid the molecular layer, the dendritic region of the granule cells. However, in the CA1 area, pyramidal cell (CA1PC) local axon collaterals extensively arborize in the stratum oriens where they are intermingled with CA1PC basal dendrites. With hundreds of boutons on the local collaterals, there is ample opportunity to form synapses with other CA1PCs. Using electron microscopy (EM), it has been shown that every third bouton of these collaterals innervates CA1PC spines (*7*), indicating that a single CA1PC can establish synaptic connections with up to 200 other CA1PCs (*8*). However, experimental data on the functional properties of this connection are unexpectedly sparse, in contrast to the wealth of information about CA1PC connections onto local GABAergic INs (*8*–*16*).

The existence of functional connections between CA1PCs was already suggested in the 1970s using antidromic stimulation of fibers that produced excitatory postsynaptic potentials (EPSPs) in CA1PCs (*17*). These experiments provided indirect evidence because orthodromic stimulations of fibers could not be excluded. Another line of evidence came from Christian and Dudek (*18*) who recorded EPSPs from CA1PCs upon glutamate microapplications onto other CA1PCs. Initial studies with dual CA1PC recordings failed to show connectivity (*19*), despite a large number of connections tested. Later, Thompson and colleagues (*20*, *21*) recorded from almost a thousand CA1PC-CA1PC pairs and detected a handful of synaptically connected pairs. The recorded postsynaptic responses displayed short-term depression (STD), suggesting high synaptic vesicle (SV) release probability (Pv) between the coupled CA1PCs. Since then, few studies examined the functional properties of this connection, but all of them used indirect methods (*22*–*25*). A recent study using retrograde rabies viruses to map the presynaptic partners of single CA1PCs demonstrated that ∼10% of the presynaptic cells of a CA1PC are other CA1PCs (*26*). This anatomical observation is in line with the results of in vivo imaging in behaving animals, showing that place field formation in a single CA1PC induces novel place fields within the same location in neighboring CA1PCs, consistent with their monosynaptic interaction through plastic synapses (*26*). Despite the convincing anatomical evidence indicating substantial connections among CA1PCs, most electrophysiological studies found either no or a very low probability of CA1PC-CA1PC connection. However, the low probability of finding CA1PC-CA1PC connections with paired recordings in acute slices does not necessary mean that these connections are negligible (*26*), because in the classical recurrent network of the CA3 area, the probability of finding CA3PC-CA3PC connections with paired recordings is also very low (*1*).

Arguably, the most direct way of demonstrating the existence of and studying the functional properties of monosynaptic connections between two nerve cells is performing dual whole-cell recordings, followed by post hoc light microscopy (LM) and EM visualization of the synaptic contact(s) between the recorded cells. Such an experimental approach has been used for decades and contributed to our understanding of the biophysical properties of central synapses and the molecular mechanisms underlying, e.g., SV release. However, studying synaptic interactions with paired recordings in circuits where the connection probability is low and the cell bodies of the interconnected cells are at large distances produces very low success rates. Alternative approaches, such as optical voltage or glutamate imaging (*27*–*37*), might yield a much larger dataset allowing more reliable functional characterization of the connections.

Here, we aimed to perform an unbiased functional examination of a large population of CA1PC local output synapses using the fluorescent glutamate indicator iGluSnFR3 and two-photon (2P) microscopy in acute slices of adult mice. This sensor, compared to previous variants, has rapid, nonsaturating activation kinetics and higher signal-to-noise ratio (*33*), making it an ideal tool to examine glutamate release from single hippocampal boutons, which usually have a single active zone and only few release sites. iGluSnFR3-expressing CA1PCs were whole-cell recorded and filled with Alexa Fluor 594, allowing us to trace their axons and image every bouton in an unbiassed manner. Using correlated EM, we identified the postsynaptic targets of imaged boutons and found that a quarter of them established synapses with CA1PC spines. Our data revealed a low Pv in these boutons: 88% of them did not release glutamate upon a single action potential (AP), 40% failed to release upon three APs, and approximately quarter of them failed to release for a complex AP train consisting of nine APs. We also show that the lack of glutamate release from these silent boutons is the consequence of improper SV priming and fusion because they can be unsilenced with the phorbol ester analog phorbol 12,13-dibutyrate (PDBU) and elevated extracellular [Ca^2+^]_e_.

## RESULTS

### Variability in glutamate release from individual CA1PC boutons

To measure glutamate release from individual CA1PC boutons, we virally expressed the glutamate sensor iGluSnFR3 in a sparse population of CA1PCs in the dorsal hippocampus of adult mice (see Materials and Methods). Sensor-expressing CA1PC somata were identified under 2P illumination and targeted for patch-clamp recordings (Fig. 1A). CA1PCs were filled with Alexa Fluor 594 (for 60 min), enabling the identification of a large number of boutons (∼20 per cell) on their local axon collaterals up to ∼400 μm from the soma (Fig. 1, A and B). Glutamate release was induced with a complex train of APs (three APs at 20 Hz, five APs at 80 Hz, and one recovery AP at 150 or 200 ms) evoked with somatic current injections in CA1PCs (Fig. 1C). The peak amplitudes of the glutamate transients displayed substantial variability across the imaged boutons (Fig. 1C and fig. S1, A and B). Within an individual CA1PC (e.g., cell shown in Fig. 1C), glutamate release for the first AP could be detected only in 15 out of 45 boutons. In 11 of the 15 boutons, the second AP evoked smaller glutamate transients, characteristics of STD (Fig. 1C, orange), whereas in the remaining 4 boutons, the transients showed short-term facilitation (STF; Fig. 1C, cyan). Most of the boutons that failed to release glutamate for the first AP (30 of 45) released for the subsequent APs (23 of 30), rendering them also as facilitating. However, seven boutons in this cell entirely failed to release glutamate for nine APs and were termed as “failures” (Fig. 1C, gray). When the traces were grouped into releases and failures and averaged, the releasing events on average showed STD [paired-pulse ratio (PPR) = 0.56 at 20 Hz]. When the depressing and facilitating transients were averaged separately, the first AP-evoked peak amplitude of depressing transients was much larger than that of the facilitating ones (Fig. 1D).

**Fig. 1.**
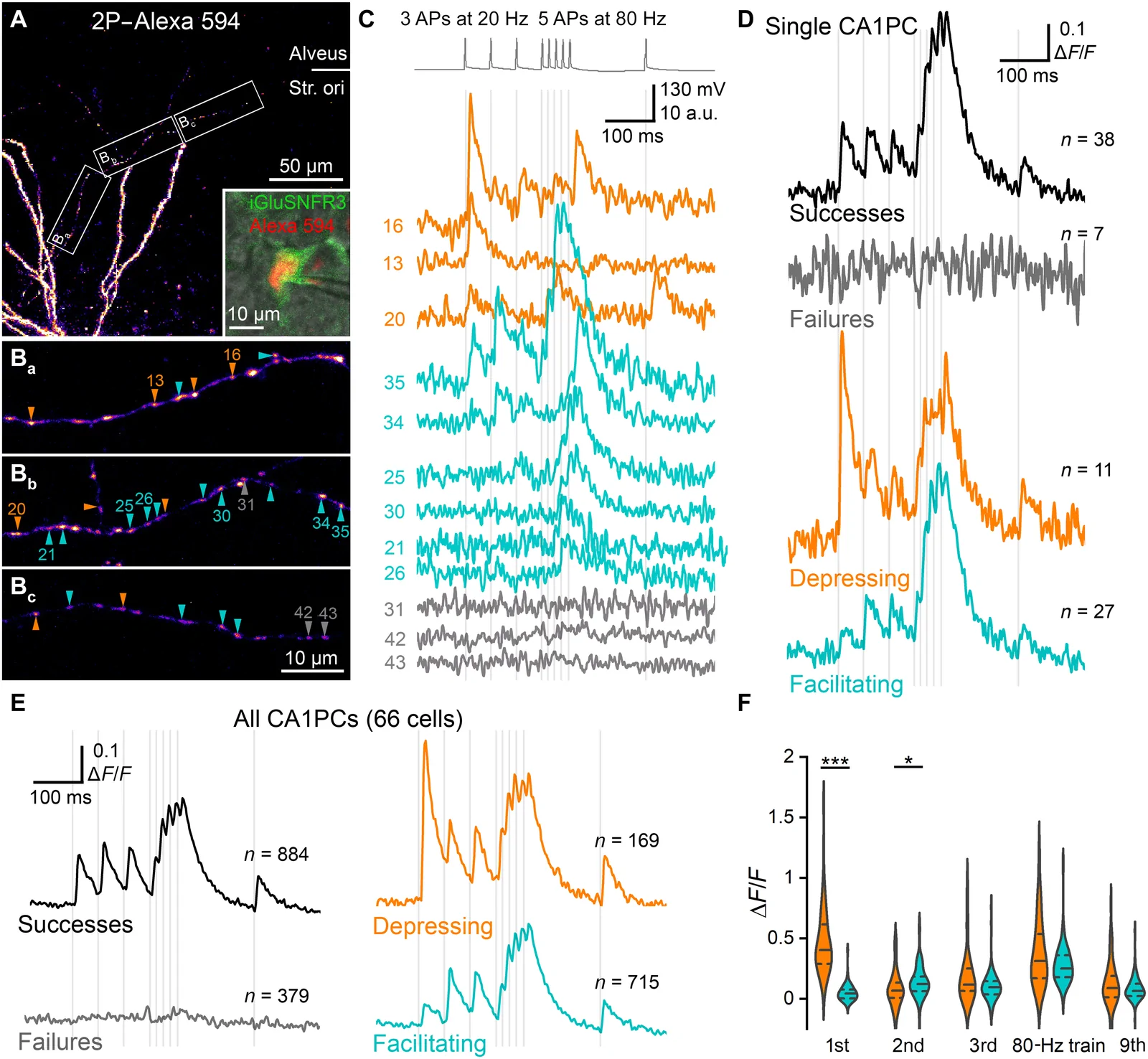
AP-evoked glutamate transients in individual CA1PC boutons display variable peak amplitudes, STP patterns, and release failures. (**A**) Low-magnification maximum intensity projection (MIP) 2P image of the basal dendritic tree and the local axon collaterals (white boxes) of a CA1PC filled with Alexa Fluor 594 (Alexa 594). Inset: Superimposed differential interference contrast and fluorescent images of the patched CA1PC soma that expresses iGluSnFR3 (green; Alexa Fluor 594, red). (**B**_**a**_ to **B**_**c**_) High-magnification MIP 2P images of the axon shown in (A). Arrowheads label boutons that were scanned. Colors correspond to different short-term plasticity (STP) phenotypes (depressing, orange; facilitating, cyan; silent, gray). Numbers correspond to traces shown in (C). (**C**) Complex AP train (three APs at 20 Hz, five APs at 80 Hz, and one AP after 200 ms) (top row) either evokes (depressing, orange traces; facilitating, cyan traces) or fails to evoke (gray traces) detectable fluorescent transients. Single traces are shown. (**D**) Averaged traces of all imaged boutons after sorting them into successes (black) and failures (gray). Successes are further grouped into depressing (orange) and facilitating (cyan) categories. Numbers of averaged traces are indicated. (**E**) Same as (D), but for all 66 PCs. (**F**) Normalized iGluSnFR3 fluorescent transient peak amplitudes during the APs train. Values are averages of depressing and facilitating transients of a given cell (*n* = 56 and 66 cells for depressing and facilitating, respectively; *P* = 2.98 × 10^−16^, 0.003, 0.08, 0.17, and 0.36, Mann-Whitney *U* test). Str. ori, stratum oriens. *P < 0.05, ***P < 0.001.

When release was examined in a total pool of 1263 boutons of 66 CA1PCs (*n* = 57 mice), we could detect glutamate release in only ∼20% (*n* = 245) of the boutons for the first AP, ∼40% for the first three APs (*n* = 507), and ∼30% showed release only upon high-frequency AP stimulations (*n* = 377, five APs at 80 Hz). Last, ∼30% of the boutons were silent (*n* = 379). The averaged glutamate transient from all releasing boutons displayed STD (PPR = 0.92 ± 0.69, *n* = 43 cells; Fig. 1E). The peak amplitude of the glutamate transients for the first AP was significantly larger for the depressing, while that of the second AP was larger for the facilitating transients (Fig. 1F). There was no significant difference in the amplitude of the fluorescent transients for all subsequent APs. The proportions of boutons showing STF (0.53 ± 0.16), STD (0.15 ± 0.11), and failures (0.32 ± 0.17; *n* = 66 cells) were similar when it was calculated from all CA1PCs and when they were derived from cells in which >20 boutons were sampled (*n* = 34 cells; fig. S1C).

When CA1PCs were grouped into deep, middle, and superficial based on the somatic location within the PC layer, the first AP-evoked peak amplitude and the sum of all peak amplitudes were not significantly different, but there was a significantly larger proportion of nonreleasing boutons in the deep (0.49 ± 0.24; *n* = 10 cells) versus superficial (0.27 ± 0.13, *n* = 39) cells (fig. S1).

### Postsynaptic IN type–dependent variability in presynaptic glutamate release

Postsynaptic target cell type–dependent difference in glutamate release in cortical and hippocampal networks is known for decades. In the hippocampal CA1 area, PCs release glutamate with low Pv when the postsynaptic cell is a somatostatin-expressing oriens lacunosum-moleculare (O-LM) IN and the postsynaptic responses display STF. When the postsynaptic target is a parvalbumin (PV)–expressing fast spiking IN (FSIN), the Pv is high, and the excitatory postsynaptic current (EPSC) shows STD. Because we saw facilitating and depressing glutamate transients from individual PC boutons, we addressed whether these could be the boutons innervating distinct IN types.

First, we selectively expressed iGluSnFR3 in O-LM INs in Chrna2-Cre mice (see Materials and Methods) and used minimal extracellular stimulations to evoke glutamate transients in O-LM IN dendrites (Fig. 2A). A complex presynaptic stimulus pattern evoked a spatially confined (<2 μm) increase in fluorescence (Fig. 2B), indicating that either one or very few neighboring synapses was stimulated. Successful release events could be detected on single traces. The peak amplitudes were much larger for the second and third compared to the first stimulus, demonstrating robust STF of release (Fig. 2, C and D). The five stimuli at 80 Hz further increased the release consistent with data obtained with paired recordings (*14*) and also demonstrate the high dynamic range of the sensor. In a separate set of experiments, we performed post hoc immunolabeling for mGluR1α following chemical fixation of the acute slices in which we had previously performed iGluSnFR3 imaging and found that boutons that synapse on mGluR1α-immunopositive INs display facilitating glutamate transients. These two lines of evidence demonstrate that some of the CA1PC boutons that display STF innervate O-LM INs.

**Fig. 2.**
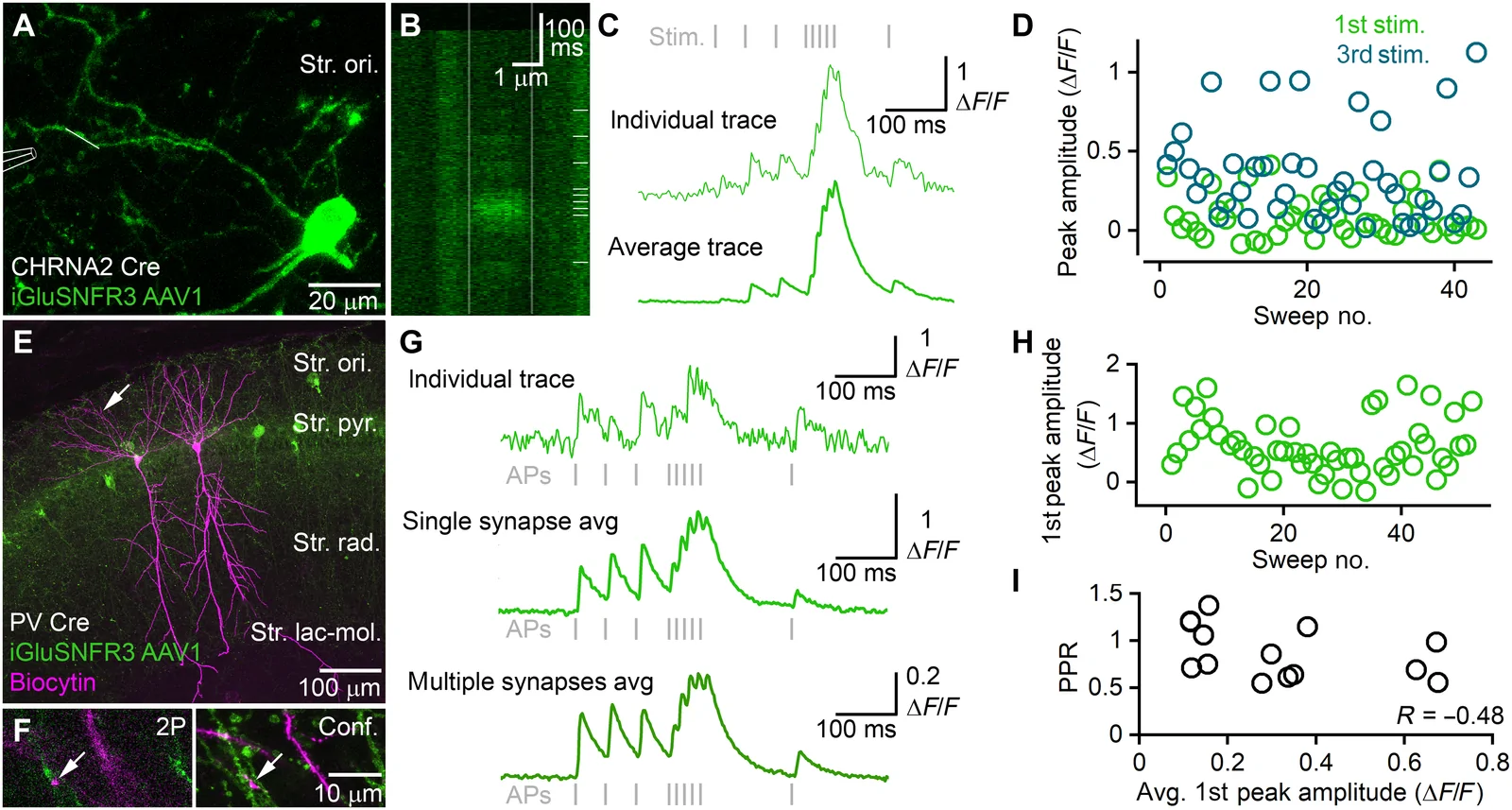
Glutamate release detected with iGluSnFR3 in O-LM and PV-expressing INs. (**A**) In vitro 2P image of an iGluSnFR3-expressing O-LM IN in a CHRNA2-Cre mouse. White line indicates the position of the line scan. (**B**) Pseudo-colored representation of the line scans across the dendritic shaft versus scanning time. Parallel short horizontal white lines indicate the stimuli. Fluorescence increased rapidly in every stimulation in a 1.5-μm confined space along the dendrite, suggesting that either one or very few synapses were activated. Vertical white lines indicate cursor positions. (**C**) Individual (top) and averaged fluorescent traces (bottom) (*n* = 44) show facilitating STP pattern. Gray vertical lines indicate the position of the stimuli. (**D**) Baseline-normalized peak amplitudes of first and third stimuli show fluctuations and stability during the imaging period. (**E**) Confocal maximal intensity projection image of iGluSnFR3-expressing PV INs [anti–green fluorescent protein (GFP) immunolabeling, green] and two CA1PCs that were filled with biocytin (magenta, visualized with Cy3-conjugated streptavidin). Arrow point to the imaged bouton of the left CA1PC. (**F**) High-magnification 2P (left) and confocal (right) images of the imaged bouton that was in close apposition to a green dendrite. Left: Alexa Fluor 594, magenta; iGluSnFR3, green. Right: Cy3-conjugated streptavidin, magenta; anti-GFP immunolabeling, green. (**G**) Individual (top) and averaged (middle: averages from 52 traces in one cell; bottom: 14 synapses from nine cells) fluorescent traces recorded from PV INs are shown. (**H**) Baseline-normalized peak amplitudes of the first AP of the train show quantal fluctuations and stability during the imaging period. (**I**) The ratios of the first and second glutamate transient amplitudes (PPR) in 14 synapses between CA1PC and PV INs show mostly STD (mean, 0.88 ± 0.28). PPR displays a slight negative correlation with the first peak amplitude (*R*, Spearman’s rank correlation coefficient; *P* = 0.08).

Next, we selectively expressed iGluSnFR3 in PV-expressing INs in PV-Cre mice (Fig. 2E). Since these INs receive a high density of excitatory input on their dendrites, extracellular stimulations often resulted in “compound,” spatially extended responses from many neighboring synapses. To record glutamate transients in PV IN dendrites evoked by a single bouton, we opted for a hybrid optical-electrical “paired recording” by whole-cell recording a CA1PC and searching for a bouton that is in close apposition to a sensor-expressing dendrite (Fig. 2F, left). A complex train of APs was then evoked with somatic current injections in the presynaptic CA1PC, and the glutamate release was detected on the postsynaptic dendrite just opposite to the bouton. All of these synapses showed high Pv, with only a limited number of failures for the first AP (Fig. 2, G and H). The average PPR of the optical signal was 0.88 ± 0.28, similar to that obtained with whole-cell paired recordings for this connection under similar Pv condition (*13*, *14*, *16*). These results demonstrate that optical glutamate imaging also reveals the postsynaptic IN type–dependent variability in presynaptic release and short-term plasticity (STP) similar to that observed with paired whole-cell recordings and that local CA1PC boutons with depressing glutamate release innervate PV-expressing FSINs.

### EM reveals the identity of the postsynaptic partners of iGluSnFR3 imaged boutons

To uncover the identity of the postsynaptic partners of the imaged CA1PC boutons, we performed correlated LM and EM analyses of more than 100 boutons of seven PCs (Figs. 3 and 4). This selected bouton population was representative of the whole population with respect to their proportion as being silent, showing STD or STF (0.27, 0.14, and 0.59, respectively). The intracellular solution contained biocytin for post hoc LM and EM visualization of the imaged boutons (Fig. 3A). Following functional imaging, the tissue was fixed and resin embedded, and bouton strings were resectioned at 100 nm (>800 consecutive sections per cell) for scanning EM (SEM). We three-dimensional (3D) reconstructed 65 biocytin-filled boutons and their postsynaptic elements for function-structure analysis (Fig. 3, B to E). Some of the boutons targeted dendrites running parallel with the alveus in the stratum oriens, receiving relatively few other synapses, a characteristic feature of O-LM IN dendrites. The glutamate transients of these boutons showed robust STF (Fig. 3B_c_). Some other boutons targeted dendrites that run perpendicular to the PC layer and were densely covered by excitatory synapses, a typical feature of PV-expressing IN dendrites. Fluorescent transients at these boutons displayed high initial amplitudes and STD (Fig. 3C_c_). We also found IN dendrites without these characteristic ultrastructural features as postsynaptic targets of functionally silent boutons (Fig. 3D_c_). Of a total of 48 IN dendrite–innervating boutons, 60% was facilitating, 25% was depressing, and 15% was silent (Fig. 3E). The averaged fluorescent transients from all IN dendrite–innervating boutons displayed STD (PPR = 0.88; Fig. 3F), and the amplitude of the fluorescent transient to the first AP was significantly higher for the depressing compared to the facilitating events (Fig. 3G).

**Fig. 3.**
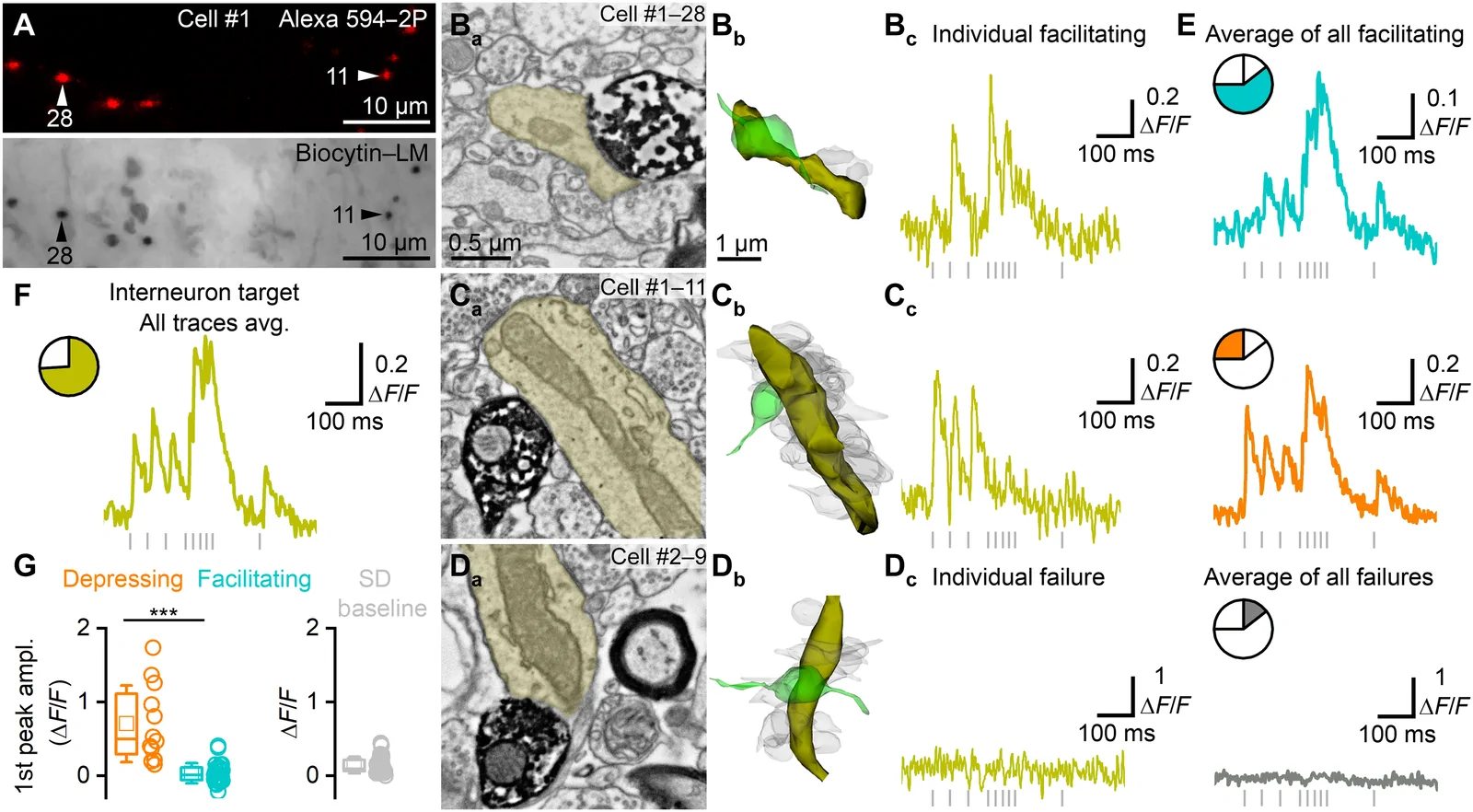
Diverse properties of glutamate transients in IN dendrite–innervating boutons. (**A**) A local axon collateral of an iGluSnFR3-expressing CA1PC filled with Alexa Fluor 594 (red) in the stratum oriens. Bottom: Same axon collateral after fixation and visualization of the biocytin. Arrowheads point to boutons that have IN dendrite as postsynaptic partners. (**B**_**a**_ to **B**_**c**_) SEM image (B_a_), 3D reconstruction (B_b_), and glutamate transients (B_c_) of bouton #28 [shown in (B_a_)] synapsing on an IN dendrite [yellow shade on (B_a_) and yellow on (B_b_)]. The IN dendrite was located in the stratum oriens near the alveus and receives low density of excitatory synapses [transparent gray in (B_b_)]. The glutamate transient evoked with nine APs (gray bars represent the position of APs) shows STF. (**C**_**a**_ to **C**_**c**_) Same as in (B) for bouton #11. The postsynaptic dendrite runs perpendicular to the stratum oriens and receives a high density of excitatory synapses. The glutamate transient shows STD. (**D**_**a**_ to **D**_**c**_) Same as in (B) for bouton #9 (shown in Fig. 4A). The postsynaptic smooth dendrite runs perpendicular to the stratum oriens and receives moderate density of synapses along the shaft. (**E**) Average of all facilitating (top), depressing (middle), and failure (bottom) glutamate transients (single scan per bouton) that are 60.4, 25.0, and 14.6% of the total bouton population synapsing on INs, respectively (insets: *n* = 29, 12, and 7 boutons in seven mice). (**F**) Average of glutamate transients measured in all the identified IN-targeting boutons (*n* = 48 boutons in seven mice). (**G**) Plots of peak amplitudes evoked by the first stimulus grouped into facilitating or depressing transients (*P* = 3.22 × 10^−6^, Mann-Whitney *U* test) and the values of the SD of the baseline noise that was subtracted from the peak amplitudes. ***P < 0.001.

**Fig. 4.**
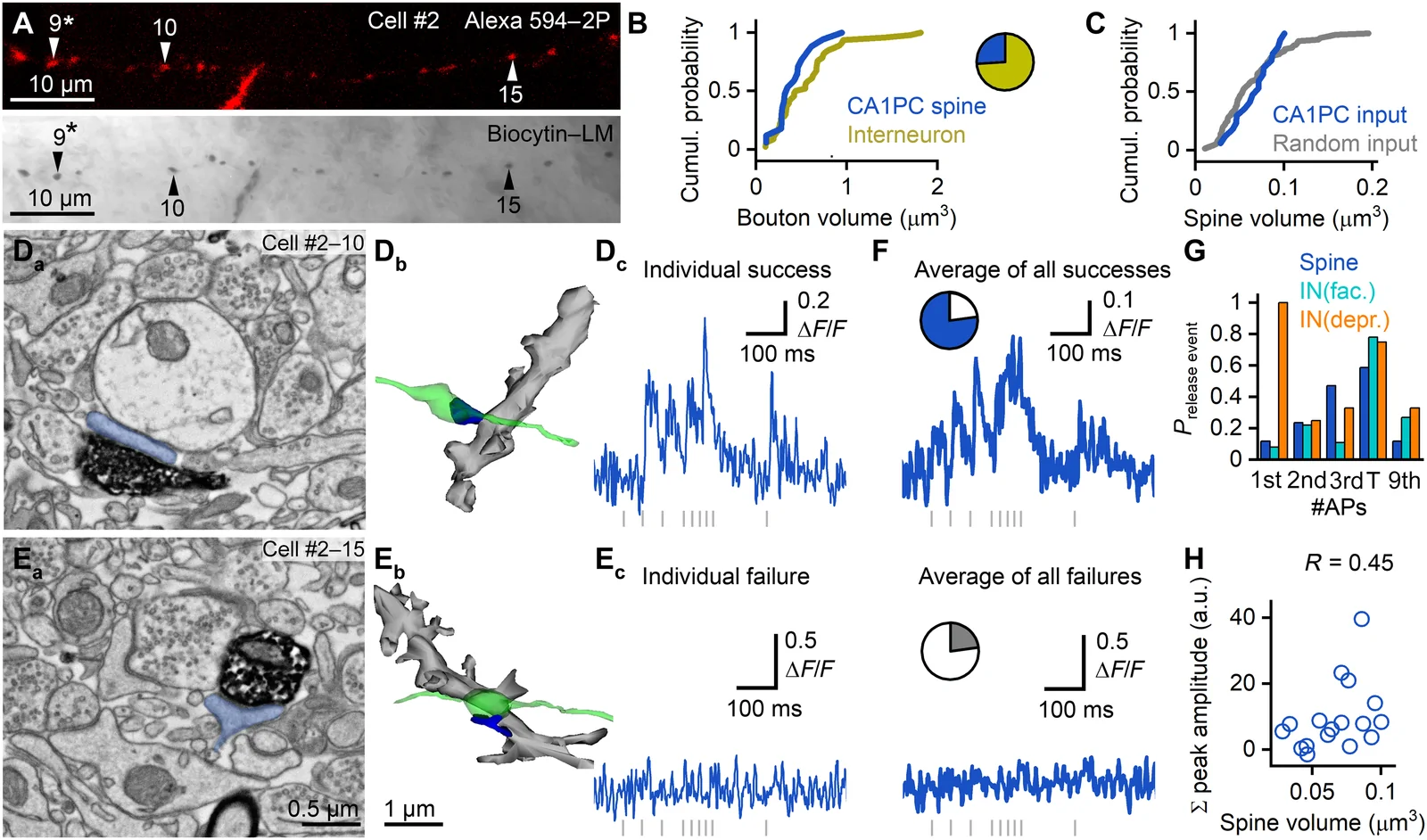
High failure rates of glutamate release in CA1PC spine–innervating boutons. (**A**) A 2P image of a local axon collateral in the stratum oriens of an iGluSnFR3-expressing CA1PC (cell #2) filled with (20 μM Alexa Fluor 594, red). Bottom: Same axon collateral after fixation and visualization of the biocytin. Boutons #10 and #15 have spines as postsynaptic partner. Bouton #9* has IN dendrite as a postsynaptic partner and is shown in Fig. 3. (**B**) Cumulative probability plot of the volume of EM identified and 3D reconstructed boutons that innervate either CA1PC spines or IN dendrites (*P* = 0.24, Mann-Whitney *U* test). Inset indicates the target distribution of these boutons (IN, 74%; spine, 26%). (**C**) Cumulative probability plot of the volume of CA1PC spines targeted by CA1PC boutons (CA1PC input, blue) and boutons of unidentified origin (gray; *P* = 0.31, Mann-Whitney *U* test). (**D**_**a**_ to **D**_**c**_) SEM image (D_a_), 3D reconstruction (D_b_), and a glutamate transient (D_c_) of bouton #10 [black on (D_a_) and transparent green on (D_b_)] synapsing on a spine [blue shade on (D_a_) and blue on (D_b_)] of a CA1PC (gray) that receives excitatory input synapses only on spines. The glutamate transient indicate successful release events upon nine APs (gray bars below the traces represent the positions of APs). (**E**_**a**_ to **E**_**c**_) Same as (D) but for bouton #15. Nine APs fail to release glutamate. (**F**) Average of all success (top) (*n* = 13 boutons) and failure (bottom) (*n* = 4 boutons) glutamate transients. (**G**) Bar graph showing the probability of release events for each APs for spine-targeting (blue), facilitating IN–targeting (cyan), and depressing IN–targeting (orange) boutons. (**H**) Relationship between the spine volume and the peak amplitude of all release events (*R*, Spearman’s rank correlation coefficient; *P* = 0.07). T, train of five APs at 80 Hz.

Our EM analysis of the postsynaptic target structures provided evidence that 26% (17 of 65) of the local boutons of CA1PCs innervate spines of other CA1PCs (Fig. 4). These boutons were intermingled with boutons targeting IN dendrites (Fig. 4A, #9* IN targeting and #10 and #15 spine targeting). Spine- and IN dendrite–innervating boutons had similar volumes and overall morphology (Fig. 4B). CA1PC spines that received innervation from identified CA1PC boutons showed a similar size distribution to a random population of basal dendritic spines that are innervated by either CA3PCs or CA1PCs (Fig. 4C). Most of the fluorescent transients at spine-innervating boutons (Fig. 4, D_a_ and D_b_) either displayed STF (Fig. 4, D_c_ and F, top trace) or were totally silent (Fig. 4, E_a_ to E_c_ and F, bottom trace). Only 12% of the spine-innervating boutons released glutamate for the first AP, 24% released glutamate for the second AP, and, even after three APs, 40% of them lacked release. Approximately quarter of the boutons did not release during the entire presynaptic AP train. Together, spine-targeting boutons showed an average low Pv and STF (Fig. 4G). Larger spines harbor larger postsynaptic density and corresponding active zone with more release sites that could potentially result in more SV release during a train of APs. However, we could find only a weak correlation between spine volume and the total amount of released glutamate (Fig. 4H), indicating that the mechanisms that restrict release are independent of the number of release sites.

### Increasing SV priming and fusion turns silent into functional boutons

To understand the mechanism behind the lack of release from a substantial fraction of boutons, first, we tested the roles of presynaptic G protein–coupled receptors. After recording a baseline period from a bouton string, a cocktail of presynaptic receptor blockers {1 μM CGP52432 [γ-aminobutyric acid type B (GABA_B_) receptor], 1 μM Ly341495 (group II mGluRs), 100 nM CGS15943 (nonspecific adenosine receptor), and 100 nM DPCPX (A1 adenosine receptor)} was washed into the bath, and the boutons were reimaged. There was no significant effect of this cocktail either on the peak amplitude of the first AP-evoked responses or on the sum of nine AP-evoked peak amplitudes (fig. S2), demonstrating that these presynaptic receptors play no role in silencing.

A number of recent studies pointed to the importance of SV priming as a key mechanisms of controlling glutamate release, and our previous work demonstrated that PDBU preferentially enhances EPSCs in O-LM INs (*13*, *38*) through increased SV priming. We set up pharmacological experiments in which we increased the fusion probability of SVs by elevating the [Ca^2+^]_e_ to 6 mM and increased SV priming with 1 μM PDBU (6Ca_PDBU). As a control experiment, we imaged the same boutons without washing in any drug and found no significant change (no rundown) in the amplitude of the fluorescent transients (Fig. 5, A and B). In contrast, 6Ca_PDBU caused a ∼5-fold increase in the response amplitude to the first AP and ∼2-fold increase on the summed amplitudes during the entire stimulus train (Fig. 5, A and B). The application of 6Ca_PDBU turned the initially silent boutons into releasing ones (Fig. 5, C and D). In a separate group of CA1PCs (*n* = 6), we repeated these experiments in a way that fluorescent transients were measured twice under control condition, which was followed by 6Ca_PDBU application (fig. S3). These experiments confirm that just waiting 15 min in control artificial cerebrospinal fluid (ACSF) does not increase release, but the subsequent application of high [Ca^2+^]_e_ and PDBU markedly and significantly increases release. These pharmacological experiments provide compelling evidence that the molecular machinery required for AP-evoked SV release is intact in silent boutons, but the Pv is so low that they appear functionally silent for ∼10 APs.

**Fig. 5.**
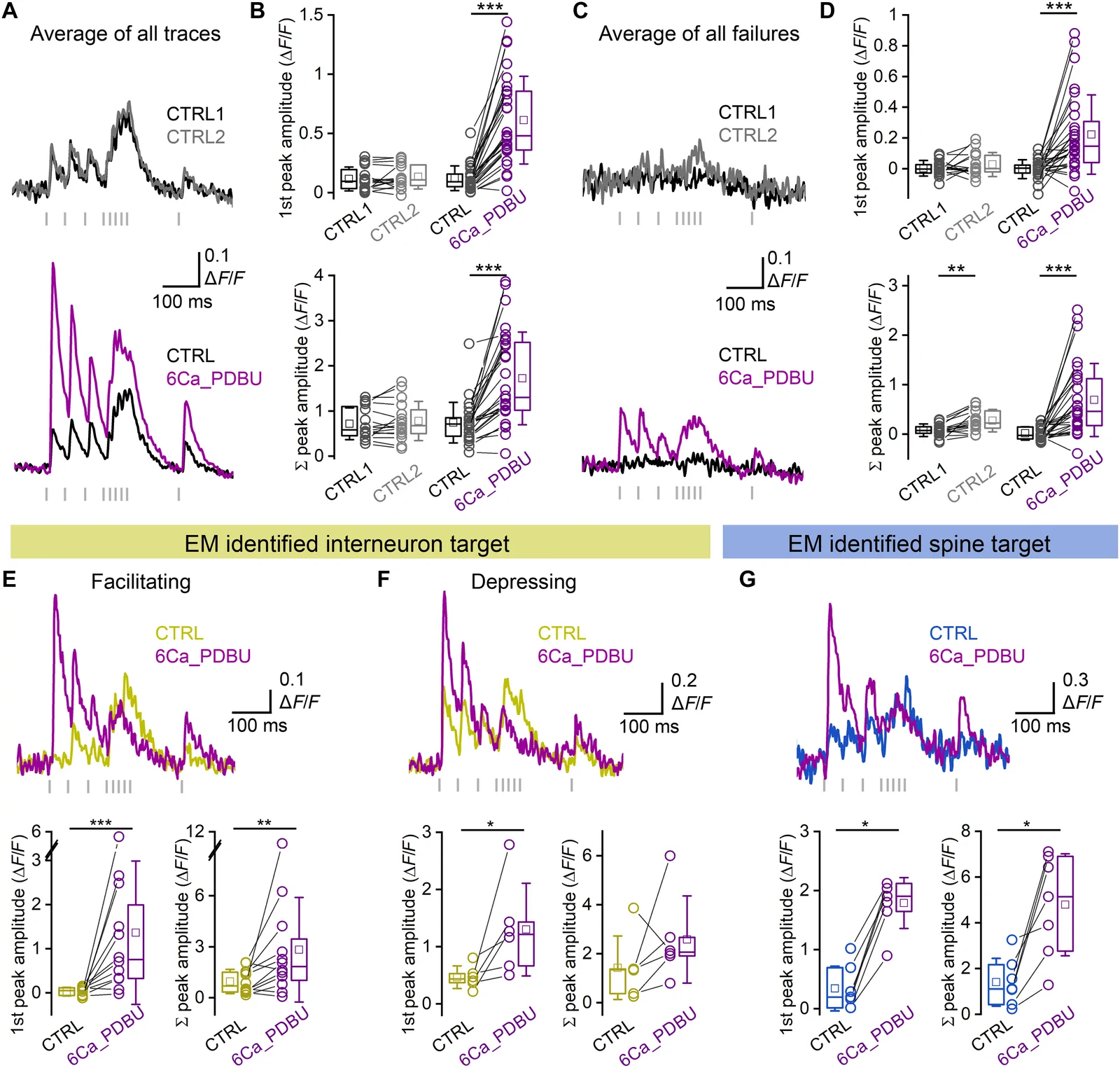
Effect of PDBU and increased [Ca^2+^]_e_ on glutamate transients. (**A**) Averaged glutamate transients under control condition (black) and either in nACSF (top) (CTRL2, gray, *n* = 16 cells, 223 boutons, 12 mice) or in nACSF containing 6 mM [Ca^2+^] and 1 μM PDBU (6Ca_PDBU, bottom) (magenta, *n* = 28 cells, 563 boutons, 22 mice). Gray bars indicate the APs. (**B**) Six millimolars of [Ca^2+^]/PDBU significantly increases the peak amplitudes evoked by the first AP (top) and the sum of peak amplitudes (bottom). Each data point is an individual cell. Wilcoxon signed-rank test (*P* = 0.56 and 1.5 × 10^−8^ for the first and *P* = 0.56 and 2.63 × 10^−5^ for the Σ). (**C**) Same as in (A) but failure transients in control are sub-selected (top: 15 cells, 60 boutons, 11 mice; bottom: 28 cells, 168 boutons, 22 mice). (**D**) The effect of 6 mM [Ca^2+^]/PDBU on failure glutamate transients. Each data point is an individual cell. Wilcoxon signed-rank test (*P* = 0.43 and 8.6 × 10^−5^ for the first and *P* = 0.003 and 2.2 × 10^−5^ for the Σ). (**E**) Averaged facilitating glutamate transients in IN-targeting boutons in control and in 6 mM [Ca^2+^]/PDBU (*n* = 12 boutons in three cells, three mice). Each data point is an individual bouton. (*P* = 9.8 × 10^−4^ for the first and *P* = 0.007 for Σ, Wilcoxon signed-rank test). (**F**) Same as in (E) for fluorescent traces that show STD (*n* = 6 boutons in three cells, three mice; *P* = 0.031 for the first and *P* = 0.31 for the Σ, Wilcoxon signed-rank test). (**G**) Averaged fluorescent traces recorded from PC spine–innervating boutons before and after the application of 6 mM [Ca^2+^]/PDBU (*n* = 7 boutons in three cells, three mice; *P* = 0.016 for the first and *P* = 0.016 for Σ, Wilcoxon signed-rank test). *P < 0.05, **P < 0.01, ***P < 0.001.

### Differential effect of 6Ca_PDBU on CA1PC boutons that innervate distinct INs

A previous study from our laboratory demonstrated that PDBU has a much larger effect on CA1PC–O-LM IN compared to CA1PC-FSIN synapses (*13*). In a final set of experiments, we tested the effect of 6Ca_PDBU on glutamate transients recorded from boutons that innervated EM-identified IN dendrites and displayed different STP. We found that 6Ca_PDBU markedly increased the first AP-evoked peak amplitude (from 0.03 ± 0.10 to 1.36 ± 1.62 Δ*F*/*F*) and the sum of amplitudes during the train for facilitating boutons to such an extent that the average output of the boutons showed STD (Fig. 5E). The same treatment resulted in only a <3-fold increase in the amplitude of glutamate transients at boutons that displayed initially STD (from 0.47 ± 0.20 to 1.30 ± 0.81 Δ*F*/*F*; Fig. 5F). The amplitude of fluorescent transients during the 80-Hz AP firing was already smaller in 6Ca_PDBU compared to control, resulting in no significant change in the amplitude of the sum of nine AP-evoked transients (Fig. 5F). Last, we examined the effect of 6Ca_PDBU on glutamate transients recorded from boutons that innervated EM-identified CA1PC spines. Similar to the effect on IN-innervating boutons that display STF, there was a robust, significant increase in transient peak amplitudes in the spine-innervating subpopulation of boutons (from 0.34 ± 0.34 to 1.79 ± 0.80 Δ*F*/*F* for the first AP and from 1.4 ± 0.85 to 4.79 ± 2.25 Δ*F*/*F* for the sum of amplitudes; Fig. 5G).

## DISCUSSION

Our present work using iGluSnFR3 imaging of individual CA1PC local output synapses revealed a robust, postsynaptic target cell type–dependent variability in presynaptic Pv and STP patterns. We confirm that parvalbumin IN–targeting boutons have high initial Pv and the release shows STD. Glutamate transients that show STD can be moderately elevated with increased [Ca^2+^]_e_ and PDBU. In contrast, O-LM IN–targeting boutons have low initial Pv, and the release displays STF. The amplitudes of the facilitating transients are markedly increased by 6 mM [Ca^2+^]_e_ and PDBU. Some of the boutons that innervate unidentified IN dendritic shafts totally failed to release glutamate upon the entire presynaptic AP train. Our EM analysis revealed that a quarter of the local CA1PC boutons innervates CA1PC basal dendritic spines, in line with the results of a previous EM study (*7*). Almost 90% of the CA1PC spine–innervating boutons did not release glutamate for a single AP, and ∼40% failed to release upon three APs at 20 Hz. One-quarter of them did not release at all during a nine-AP presynaptic stimulus train. Our post hoc EM analysis demonstrated that these “failure” boutons had normal ultrastructure and were indistinguishable from those that released glutamate. Our EM analysis indicated that these boutons should be capable of normal SV release, but our pharmacological experiments provided direct evidence that the molecular machinery necessary for SV release is intact in these “silent” boutons and can be activated by PDBU/high [Ca^2+^]_e_. These results imply that when CA1PC-CA1PC connections are probed with few APs (used in most physiological studies), most of them would be categorized as uncoupled.

The concept of silent synapses has been around for decades, which is supported by experimental data obtained from different organisms, brain areas, and cell types. In the rodent hippocampus, silent Schaffer collateral–to–CA1PC spine synapses have been described over three decades ago, which harbor only postsynaptic *N*-methyl-d-aspartate (NMDA) but not AMPA receptors (*39*–*43*). As a consequence, under physiological conditions, they do not conduct current due to a voltage-dependent Mg^2+^ block of the NMDA receptors. Thus, these synapses are considered as postsynaptically silent. Induction protocols of long-term potentiation (LTP) result in insertion of postsynaptic AMPA receptors leading to the “unsilencing” of these synapses (*42*).

Early evidence for presynaptically silent synapses was based on findings that the number of functional release sites was smaller than the number of synaptic contact sites between two neurons (*44*). Subsequent studies using fluorescent styryl dyes such as FM1-43 to monitor SV release demonstrated that many post hoc–identified synapses lacked FM1-43 labeling after intense presynaptic activity and concluded that they were presynaptically silent or “dormant” (*45*–*47*). The proportion of these dormant synapses can be altered with long-lasting changes in neuronal activity (*48*, *49*), indicating its role in homeostatic plasticity. Pharmacological manipulations that affect Munc13-1 function provide an effective way to “unsilence” these synapses (*50*). It has also been shown that Hebbian LTP induction protocols can unsilence presynaptically silent synapses, leading to an apparent increase in the number of release sites (*51*).

Imaging SV release with FM dyes is a powerful technique, but it cannot be targeted to defined cell types and apparently difficult to perform in acute brain slices and impossible in intact brains. The development of genetically encoded fluorescent glutamate sensors provided a long needed tool for studying glutamate release in intact circuits, including in vivo imaging in behaving animals (*28*, *31*–*34*, *36*, *37*, *52*). Glutamate sensors have the sensitivity to detect the release of single SVs (*29*, *30*, *35*), thus allowing the investigation of biophysical properties of synapses and distinguishing between silent and active synapses.

Taking advantage of the recently developed iGluSnFR3, here, we demonstrate that a significant portion (∼30%) of CA1PC boutons do not release glutamate upon a complex train of presynaptic APs and can be considered as presynaptically silent. Our data demonstrate that they contact either unidentified IN dendrites or CA1PC basal dendritic spines. Because, in our experiments, we detect all PV-like dendrite-innervating CA1PC boutons as being active, we argue that the presynaptically silent synapses cannot be a consequence of a lack of sensitivity or insufficient signal-to-noise ratio of the sensor. Thus, the lack of release is likely a biological property and is postsynaptic target cell type dependent. Our post hoc EM examination and pharmacological experiments with PDBU provide compelling evidence that these silent boutons are structurally and molecularly intact but do not harbor release competent SVs. Our pharmacological experiments with a cocktail of antagonist revealed that tonic activation of presynaptic receptors is not responsible for silencing these boutons. Rather, an insufficient vesicle priming by Munc13-1 is a main reason for the lack of SV release, which is consistent with studies examining SV release from cultured neurons (*50*). It remains to be determined how and under what natural activity conditions can these synapses be unsilenced. Furthermore, as the relationship between pre- and postsynaptically silent synapses is unknown, future experiments with post hoc localization of AMPA receptors will be needed to determine whether the presynaptically silent synapses harbor or lack postsynaptic AMPA receptors.

CA1PCs residing in different sublayers differ in their molecular composition (*53*) and in many functional properties, including theta and gamma phase preference, firing rates, bursting propensity, and the proportion of spatially tuned cells (*54*). Here, we found that they also differ in the proportion of their silent local output synapses, suggesting that they are differentially integrated in the local circuitry. This difference in circuit connectivity has been previously described, revealing differential reciprocal connectivity with INs (*8*, *11*).

Previous studies from our laboratory using paired patch-clamp recordings have demonstrated that the so-called postsynaptic target cell type–dependent variability on Pv and STP in the hippocampal network is primarily the consequence of differential SV priming at CA1PC-FSIN versus CA1PC–O-LM IN synapses (*13*, *14*, *16*). Here, using glutamate imaging, we not only confirm our previous results but also extended it to other postsynaptic target cell types. Our EM examination indicates that some yet unidentified IN dendrites receive silent synapses. Furthermore, CA1PC-CA1PC synapses also behave differently from CA1PC-FSIN and CA1PC–O-LM IN synapses. From these results, we conclude that every postsynaptic target cell type dictates specific release properties to their presynaptic boutons, suggesting that the postsynaptic target cell type–dependent differences in presynaptic release is a general principle in the organization of the hippocampal network.

Local connectivity of CA1PCs is considered to be negligible, leading to the notion that CA1PCs—unlike CA3PCs—do not form a recurrent network. This is in contrast to the findings of an EM study demonstrating that 30% of the local CA1PC boutons innervate CA1PC spines (*7*), which is in line with our EM data. The scarcity of finding functionally connected CA1PCs with multiple electrical recordings is not a strong evidence for the lack of these connections because in the classical recurrent network of the CA3 area, the probability of finding connected pairs is also rather low (∼1%) (*1*, *55*). This low connectivity is in contrast with a 10-fold larger connection probability as estimated from large volume EM reconstructions (*56*). Using voltage imaging, Layous *et al.* (*27*) found a higher (∼5%) connectivity rate among CA3PCs compared to paired recordings, suggesting that electrophysiological experiments consistently underestimate connection probabilities. Our present glutamate imaging data demonstrate that only a very small fraction of the CA1PC-CA1PC connections has substantial initial Pv and, if only these connections are detected with paired recordings, it could explain the discrepancy between the above reported connection probabilities.

A recent study using in vivo [Ca^2+^] imaging in a large population of CA1PCs in head-restrained behaving mice demonstrated that the activation of a single “seed” CA1PC can initiate an increased activity in some other CA1PCs (*26*). The recruited CA1PCs were more likely to form a place field in the location where the seed neuron was active, resulting in an amplification of the representation of that specific locations. By performing computational modeling, Geiller *et al.* (*26*) have also shown that a subnetwork model with synaptically interconnected CA1PCs and INs can describe the experimental data. The precise molecular mechanisms of neuronal plasticity underlying this local circuit amplification of feature selectivity is unknown, but we propose that unsilencing of presynaptically silent CA1PC-CA1PC synapses might be responsible for the emergence of CA1PC functional interconnectivity. It will be interesting to see how distinct long-term synaptic plasticity mechanisms (e.g., spike timing–dependent versus behavioral time scale plasticity) (*57*) can cause long-term changes in SV priming in these silent CA1PC synapses.

## MATERIALS AND METHODS

### Animals

Fifty-seven adult C57BL/6J [postnatal day 50 (P50) to P70], 2 adult Tg(Chrna2-Cre)OE25Gsat/Mmucd (RRID: MMRRC_036502-UCD) on C57BL/6J background (*58*), and 14 adult PV-Cre mouse (RRID: IMSR_JAX:017320) on FVB/Ant background were used. The animals were housed in the vivarium of the Institute of Experimental Medicine in a normal 12-hour/12-hour light/dark cycle and had access to water and food ad libitum. All the experiments were carried out in accordance with the Hungarian Act of Animal Care and Experimentation 40/2013 (II.14) and with the ethical guidelines of the Institute of Experimental Medicine Protection of Research Subjects Committee (license no. MÁB 4/2021).

### Virus injection

Mice were anesthetized with a mixture of ketamine, xylazine, and pypolphene (0.625, 6.25, 1.25 mg/ml, respectively, 10 ml/g of body weight). pAAV1-Ef1a-iGluSnFR3-IRES-Cre GPI (Addgene, 157181; 1.2 × 10^12^ titers) mixed with pENN.AAV9-CMVa.PI.Cre.rBG (Addgene, 105537, 5 × 10^10^) or alone (for CHRNA2-Cre and PV-Cre mice) was injected into the dorsal hippocampus (200 nl per injection site) into two coordinates from the Bregma: anteroposterior, 2.2 and 2.4 mm; dorsoventral, 1.2 and 1.4 mm; lateral, 1.5 and 1.6 mm. After 4 to 6 weeks, in vitro acute slices were cut from the dorsal hippocampus as below.

### Slice preparation and electrophysiological recordings

A similar method was used as described previously (*13*). Mice were stably anesthetized with a cocktail of ketamine, xylazine, and pypolphene (0.625, 6.25, and 1.25 mg/ml, respectively, 10 ml/g of body weight) and decapitated; the brain was quickly removed and placed into an ice-cold cutting solution containing the following: 205.2 mM sucrose, 2.5 mM KCl, 26 mM NaHCO_3_, 0.5 mM CaCl_2_, 5 mM MgCl_2_, 1.25 mM NaH_2_PO_4_, and 10 mM glucose, saturated with 95% O_2_ and 5% CO_2_. Coronal slices (250 μm) were then cut from the dorsal part of the hippocampus using a Leica Vibratome (VT1200S) and were incubated in a submerged-type holding chamber in ACSF containing the following: 126 mM NaCl, 2.5 mM KCl, 26 mM NaHCO_3_, 2 mM CaCl_2_, 2 mM MgCl_2_, 1.25 mM NaH_2_PO_4_, and 10 mM glucose, saturated with 95% O_2_ and 5% CO_2_ (pH 7.2 to 7.4) at 36°C for an hour, and then kept at 22° to 24°C until use. Recordings were performed at 32°C up to 6 hours after slicing in the same ACSF containing 2 μM AM251 to block presynaptic CB1 receptors. Cells were visualized using a Femto2D microscope equipped with an oblique illumination and a water-immersion lens (25×, numerical aperture = 1.05; Olympus). Virally infected CA1PCs and INs of the dorsal hippocampus were identified on the basis of their green soma. Whole-cell current-clamp recordings were performed from CA1PCs using a MultiClamp 700B amplifier (Molecular Devices). Recorded traces were filtered at 3 to 4 kHz and digitized online at 20 kHz. Patch pipettes (resistance, 3 to 6 MΩ) were pulled from thick-walled borosilicate glass capillaries with an inner filament. Intracellular solution contained the following: 130 mM K-gluconate, 5 mM KCl, 2 mM MgCl_2_, 0.05 mM EGTA, 10 mM creatine phosphate, 10 mM Hepes, 2 mM adenosine 5′-triphosphate, 1 mM guanosine 5′-triphosphate, 25 mM Alexa Fluor 594 (Invitrogen), 10 mM glutamate, and 7 mM biocytin (pH 7.3, 290 to 300 mosmol). CA1PCs were held at −65 mV (with a maximum of ±100-pA dc current), and single or multiple APs were evoked with 2- to 3-ms-long depolarizing current pulses (1 to 1.2 nA).

For extracellularly evoked responses, a bipolar theta stimulating electrode (filled with ACSF) were placed in the stratum oriens at a distance of ∼40 μm from a selected dendrite of the iGluSnFR3-expressing IN, and a train of stimuli (three stimuli at 20 Hz, then five stimuli at 80 Hz, and another stimulus, 200 ms later; 14 μA, 2 ms) were applied and repeated every minute (Biostim STI-4a, Supertech). CA1PCs were categorized on the basis of their soma location as superficial, middle, and deep. We classified cells as superficial if the soma was at the border of the stratum radiatum and pyramidale, as deep if at the border of the stratum pyramidale and oriens, and as middle otherwise.

### 2P laser scanning microscopy

Experiments were performed with a Femto2D (Femtonics) laser scanning microscope equipped with a MaiTai femtosecond pulsed laser tuned to 810 nm and a MaiTai Deep See femtosecond pulsed laser tuned to 920 nm. Electrophysiological data and image acquisition were controlled with a software (Mes, Femtonics). CA1PCs were filled for 60 min with intracellular solution containing 25 mM Alexa Fluor 594. Boutons were selected at distances of 150 to 400 μm from the soma (at 30 to 80 μm in depth in the tissue) using the Alexa Fluor 594 signal excited with 810 nm (laser intensity, 3 to 6 mW at the back aperture of the objective lens) and then imaged in line-scan mode (at 1 kHz, 1 per minute repetition) at 920 nm (laser intensity, 3 to 12 mW). Each bouton (on average, 24 ± 12 per cell) was scanned once for baseline measurement and once after washing in normal ACSF with AM251 (control) or a drug solution for 10 min. A complex AP train–evoked (three APs at 20 Hz, then five APs at 80 Hz, and another AP, 150 to 200 ms later) changes in fluorescence were recorded. Fluorescent traces were Gauss filtered (200 Hz), and changes in fluorescence were quantified as

$$\Delta F/F=\left[F_{\text{green}\left(t\right)}-F_{\text{baseline.green}}\right]/\left(F_{\text{baseline}.\text{green}}-I_{\text{dark}.\text{green}}\right)$$

*F*_green(t)_ is the green fluorescence signal as a function of time, *F*_baseline.green_ is the average of the green fluorescence before stimulation in a 100-ms time window, and *I*_dark.green_ is the dark current in the green channel (averaged over 20 ms). Peak amplitude of the fluorescent signal was defined as the maximum value within a 10-ms time window after the peak of the APs minus the SD of the baseline fluorescence. The maximum peak amplitude was measured for the five APs at 80-Hz train (time window between the fourth AP and 10 ms after the eighth AP). Responses for each AP were sorted into groups of failures and successes with a threshold value of 2.75× SD of the baseline.

Traces with any sign of photo damage or high baseline noise were excluded from the analyses. The following exclusion criteria were used: (i) negative baseline value; (ii) baseline value larger than the mean baseline of all cells +2× SD; (iii) SD of baseline divided by mean of baseline exceeds 0.6; (iv) bleaching exceeds 20% (bleaching defined as the mean of last 100 ms of the 700-ms trace divided by the mean of first 100 ms of the trace).

The following drugs were applied: 2 μM AM251, 1 μM PDBU, 1 μM CGP52432 (GABA_B_ receptor), 1 μM Ly341495 (group II mGluRs), 100 nM CGS15943 (nonspecific adenosine receptor), and 100 nM DPCPX (A1 adenosine receptor). All drugs were purchased from Tocris Bioscience.

### Tissue processing

After recordings, slices selected for post hoc immunolabeling were fixed in 4% formaldehyde (FA) and 0.2% picric acid in 0.1 M phosphate buffer (PB) (pH 7.4) at 4°C for 12 hours. Biocytin was visualized with Cy3-conjugated streptavidin (1:1000; Thermo Fisher Scientific). Immunolabeling was carried out using either rabbit anti–green fluorescent protein (GFP) (1:1000; Millipore, AB3080P) and Alexa Fluor 488–conjugated goat anti-rabbit (the Jackson Laboratory) antibodies (for PV-Cre animals) or Guinea pig anti-mGluR1α (1:1000; Synaptic Systems) and Alexa Fluor 488–conjugated goat anti-Guinea pig (the Jackson Laboratory) antibodies (for C57Bl6J animals).

For SEM, slices were fixed [4% FA, 0.1 to 1% glutaraldehyde, and 0.2% picric acid in 0.1 M PB (pH 7.4) at 4°C for at least 24 hours], embedded in agarose, and resectioned at 50 μm in thickness. Biocytin was visualized using an avidin–biotin–horseradish peroxidase complex (Vector Laboratories), followed by a reaction with 3′3-diaminobenzidine tetrahydrochloride as chromogen and 0.01% H_2_O_2_ as oxidant for 12 min. Sections were then fixed in 1% OsO_4_ and 0.75% potassium ferrocyanide for 30 min on ice and then for 30 min at room temperature, washed in distilled water, stained in 1% uranyl acetate for 30 min and then with lead aspartate for 2 min, dehydrated in a graded series of ethanol, and embedded in epoxy resin (Durcupan). LM image stacks were aligned with the 2P image stacks to identify the imaged boutons. Imaged axonal segments were then reembedded and sectioned at 100 nm. Ribbons of consecutive ultrathin sections (*n* = 400 to 800 sections per sample) were transferred to silicon wafers (Ted Pella) for SEM.

### Scanning electron microscopy

Sections were imaged with field-emission SEM (FEI Apreo SEM, Thermo Fisher Scientific) in backscattered mode. The micrographs were acquired with array tomography plugin in MAPs software (version 3.10, Thermo Fisher Scientific), with the following imaging parameters: 4-mm working distance at high vacuum with a beam current of 0.2 nA, a voltage of 2.5 kV, a dwell time of 5 μs per pixel, a pixel size of 3 or 8 nm (for high- and low-magnification images, respectively), and 6000 × 6000 or 8000 × 8000 pixel per image. The SEM stacks were imported into Fiji TrakEM2 plugin for fine alignment.

### Identification of boutons across 2P- light- and EM

We record the *xyz* coordinates of each imaged bouton in vitro using the 2P microscope (image stacks with 1- to 5-μm steps at high and low magnifications) and post hoc with a light microscope (0.5-μm Z-steps Zeiss axioscope equipped with 100× oil immersion objective). The coordinates were used as guides for finding the identified bouton at low-magnification SEM images in addition to using capillaries, unlabeled cell bodies, and dendrites of the labeled cells as landmarks. Identified boutons with their postsynaptic partner were 3D reconstructed using Reconstruct software (https://synapseweb.clm.utexas.edu/software).

### Data analysis and statistical tests

Analysis of electrophysiological and 2P imaging data was performed using MES (Femtonics). Data were plotted using OriginPro. Most of the data showed non-normal distribution (Kolmogorov-Smirnov test); thus, mainly nonparametric tests were used. Correlations were assessed using linear regression fit to the data (*R* indicates Spearman correlation coefficient). Statistical significance between two independent groups was assessed with Mann-Whitney *U* test. Between two dependent groups, Wilcoxon signed-rank test was used. To compare more than two groups, Kruskal-Wallis analysis of variance (ANOVA) with post hoc Dunn’s test was applied. For more than two dependent groups, Friedman ANOVA with post hoc Wilcoxon-Nemenyi-McDonald-Thompson test was used. Results were considered significant with *P* < 0.05. Data are presented as means ± SD throughout the paper, box plots show mean (square), median (line), SD (whiskers), and interquartiles (box).
