# Supplementary material for: Incomplete synaptic vesicle priming functionally disconnects CA1 pyramidal cells
Source: Sci Adv. 2026 Nov 11;12(46):eaej3501. doi: 10.1126/sciadv.aej3501 (PMC13753995; doi:10.1126/sciadv.aej3501)
Supplement: Supplementary file 1 [file sciadv.aej3501_sm.pdf]

Science Advances
AAAS

Supplementary Materials for

Incomplete synaptic vesicle priming functionally disconnects CA1 pyramidal cells

Noemi Holderith and Zoltan Nusser

Corresponding author: Noemi Holderith, noemi.holderith@koki.hu; Zoltan Nusser, nusser@koki.hu

Sci. Adv. **12**, eaej3501 (2026)
DOI: 10.1126/sciadv.aej3501

This PDF file includes:

Figs. S1 to S3

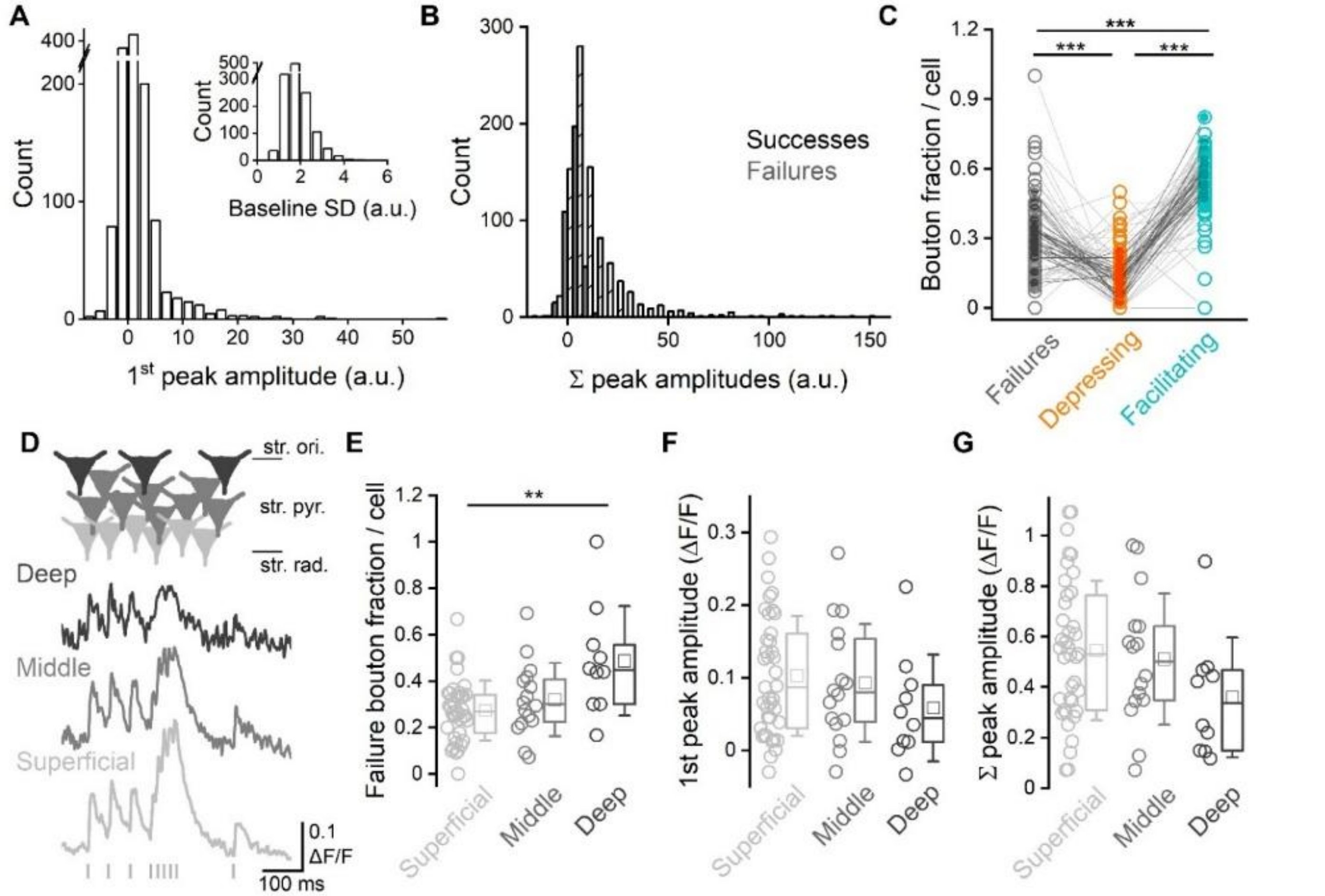

Figure S1. Heterogeneity of release properties of CA1PC boutons.

(**A**) Frequency distribution of the peak amplitudes of fluorescent transients in response to the 1st AP of the stimulus train measured in 1263 boutons in 66 CA1PCs (n = 57 mice). Values are not normalized to baseline fluorescence. Inset: distribution of the standard deviation values of the baseline noise.

(**B**) Frequency distribution of summed peak amplitudes (1-3rd + 80 Hz train + 9th) of traces exhibiting release failures (grey bars) and successes (hollow bars). Failures are defined by the lack of iGluSNFR3 transient for all of the APs throughout the train.

(**C**) Proportions of boutons displaying failures, depressing or facilitating responses in CA1PC (large symbols) are significantly different ($p = 3.3*10^{-16}$, Friedman-ANOVA, $2.2*10^{-5}$, $2.3*10^{-4}$, $9.3*10^{-17}$ *post hoc* Dunn's test). Superimposed small symbols show CA1PCs with >20 boutons imaged.

(**D**) Schematic demonstration of the position of 'superficial', 'middle' and 'deep' CA1PCs within the pyramidal cell layer. Averaged traces of all imaged CA1PCs grouped according to the soma position in the PC layer.

(**E**) Ratios of silent boutons per cell. Superficial CA1PCs have significantly smaller fraction of boutons that do not show release during a train of APs than deep CA1PCs. (Superficial, middle, deep: 0.27 ± 0.13, 0.32 ± 0.16, 0.49 ± 0.24, p = 0.011, KW-ANOVA, superficial vs deep p = 0.008 *post hoc* Dunn's test).

(**F**) Peak amplitude of the glutamate transients for the 1st AP averaged within individual cells and grouped according to the soma position in the layer.

(**G**) Same as F for the Σ peak amplitudes during the AP train.

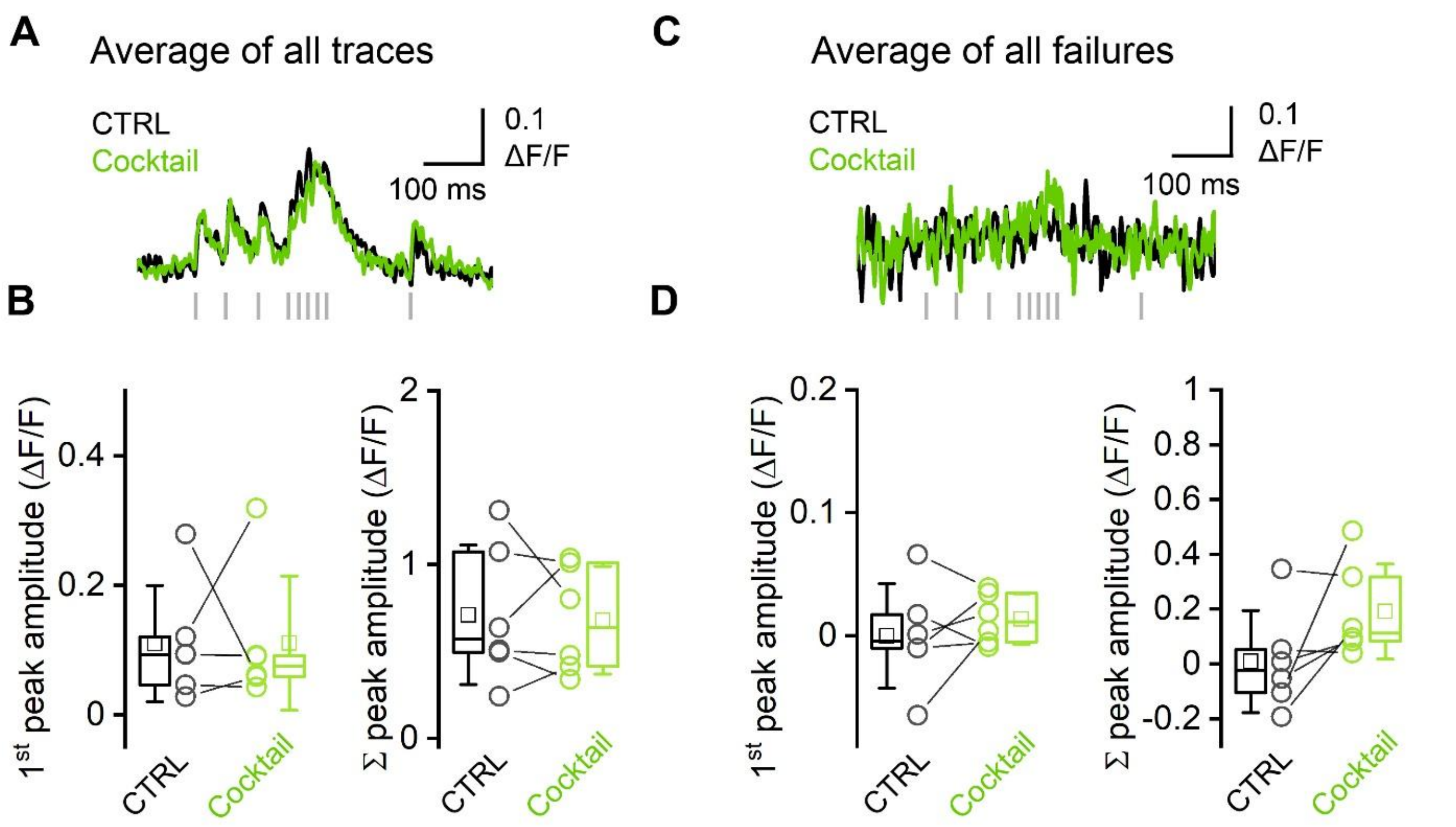

Figure S2. Pharmacological blockage of presynaptic receptors does not alter glutamate transients in CA1PC boutons

(**A**) Averaged glutamate transients evoked with a train of 9 APs in control condition (CTRL black) and following the wash in of a cocktail of presynaptic receptor blockers (1 µM CGP52432, 1 µM Ly341495, 100 nM CGS15943, 100 nM DPCPX; green, n = 6 cells, 114 boutons, 6 mice). Grey bars bellow the traces represent the position of the APs.

(**B**) A cocktail of presynaptic receptor blockers does not change the peak amplitude (left: 1st amplitude, right: sum of 9 peak amplitudes) of fluorescent transients. Each data point is an individual cell. Wilcoxon signed-rank test ($p = 0.84$ for the 1st and $p = 0.84$ for the Σ).

(**C**) Same as in (A) but failures for the 1st scan are subselected (left n = 6 cells, 35 boutons, 6 mice).

(**D**) A cocktail of presynaptic receptor blockers does not change the peak amplitude (left: 1st amplitude, right: sum of 9 peak amplitudes) of the sub-selected failure traces. Each data point is an individual cell. Wilcoxon signed-rank test ($p = 0.42$ for the 1st, $p = 0.12$ and for the Σ).

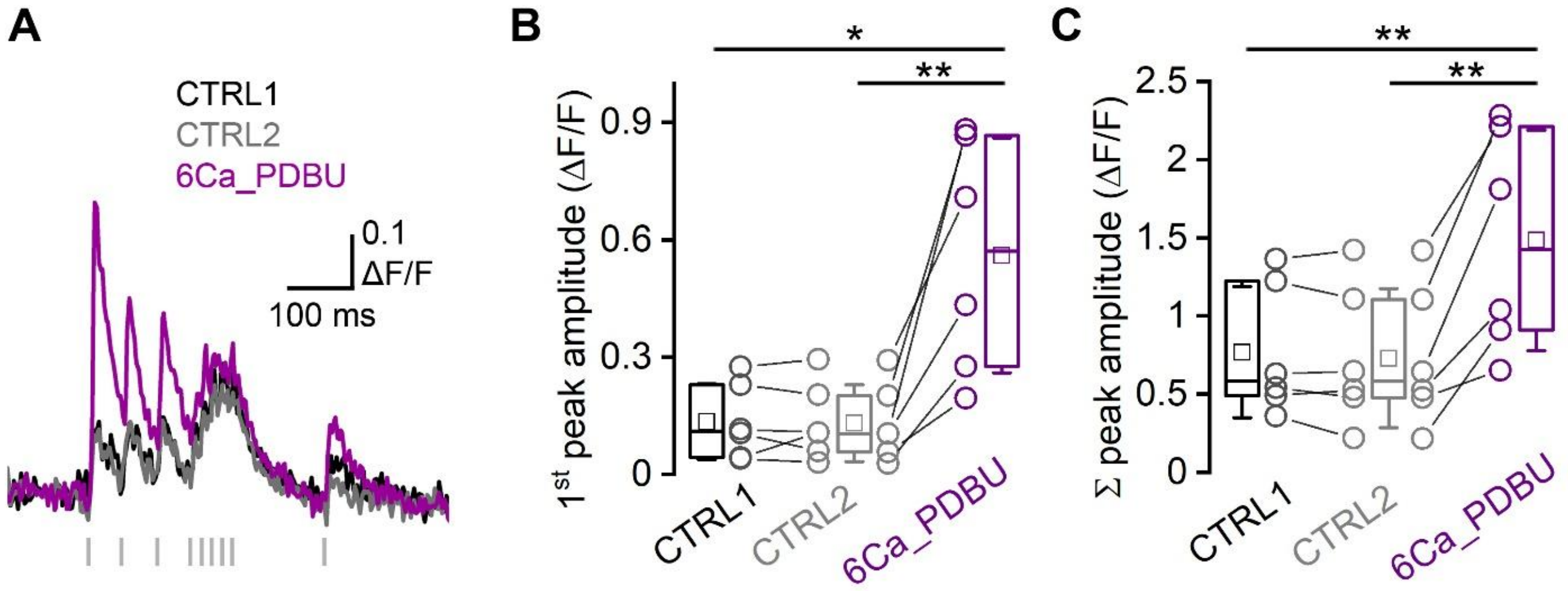

Figure S3. Effect of 1 μM PDBU and 6 mM Ca^{2+} on glutamate transients

(**A**) Averaged glutamate transients evoked with a train of 9 APs in 2 control periods (CTRL1: black, CTRL2: grey) followed by a wash in of 6 mM Ca^{2+} and 1 μM PDBU (magenta, n = 6 cells, 109 boutons, 5 mice). Light grey bars bellow the traces represent the position of the APs.

(**B**-**C**) 6mM $[Ca^{2+}]_e$ and 1 μM PDBU significantly increases the peak amplitude of the fluorescent transient for the 1st AP (B) and the sum of all 9 peak amplitudes (C) while no change was detected at repeated control (CTRL) measurements. Each data point is an individual cell. (for 1st AP-evoked amplitudes: Friedman-ANOVA, p = 0.009, *post hoc* Wilcoxon-Nemenyi-McDonald-Thompson test 0.83, 0.05, 0.01; for the Σ amplitudes: p = 0.01, *post hoc* Wilcoxon-Nemenyi-McDonald-Thompson test 1, 0.03, 0.03).
